# Supplementary material for: Differential Sensitivity of HSV-1 and PRV to IFN-λ Reveals a Neuron-Specific Antiviral Role for RSAD2
Source: bioRxiv. 2026 Jun 2:2026.06.01.729199. Preprint. [Version 1] doi: 10.64898/2026.06.01.729199 (PMC13252147; doi:10.64898/2026.06.01.729199)

bioRxiv preprint doi: https://doi.org/10.64898/2026.06.01.729199; this version posted June 2, 2026. The copyright holder for this preprint (which was not certified by peer review) is the author/funder, who has granted bioRxiv a license to display the preprint in perpetuity. It is made available under a CC-BY 4.0 International license.

Supplemental Information

Supplemental Figure 1. Differential gene expression profiles at early and late timepoints following IFN-λ treatment in SCGs. (A) Venn diagram of differentially expressed genes (DEGs) (FDR <5%) comparing early (3 h) and late (24 h) responses. Shared genes are primarily linked to the innate immune response (40%). *gProfiler* was utilized to categorize genes. *GeneVenn* was used to identify genes shared after 3 and 24 hpt with IFN-λ. (B) Table demonstrating significantly induced genes (FDR <5%) shared between 3 h and 24 hpt primary neurons (left) and uniquely enriched genes at 24 hpt (right).

A)

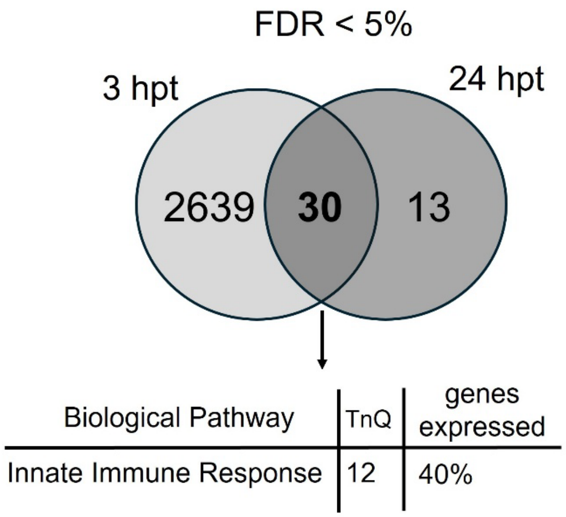

B)

FDR < 5% (Shared)	FDR < 5% (24h post-treatment)
Apol9a	Bst2
Cxcl10	Gbp2
Ddx60	Ifgga2l1
Dhx58	Ifitm3
Dtx3l	Isg15
Ifi44	Oas1a
Ifih1	Oasl
Ifit1	Oasl2
Ifit2	Psmb8
Ifit3	Slfn5
Irf7	Sp100
Irgm2	Sp110
Lgals3bp	Xaf1
Lgals9	
Mx1	
Mx2	
Oas1b	
Oas1i	
Parp14	
Parp9	
RT1-A2	
Rig1	
Rn45s	
**Rsad2**	
Samd9	
Samd9l1	
Slfn13	
Stat1	
Uba7	
Usp18	

bioRxiv preprint doi: https://doi.org/10.64898/2026.06.01.729199; this version posted June 2, 2026. The copyright holder for this preprint (which was not certified by peer review) is the author/funder, who has granted bioRxiv a license to display the preprint in perpetuity. It is made available under a CC-BY 4.0 International license.

Supplemental Figure 2. ISG expression kinetics in SK-N-SH cells. RSAD2, Oas1a and IFIT1 expressions were quantified by Q-PCR relative to GAPDH following human IFN-λ treatment for 3-, 8- and 24-hours. Statistical significance was performed using Student's *t* test: *, $p < 0.05$ (n=3 replicates).

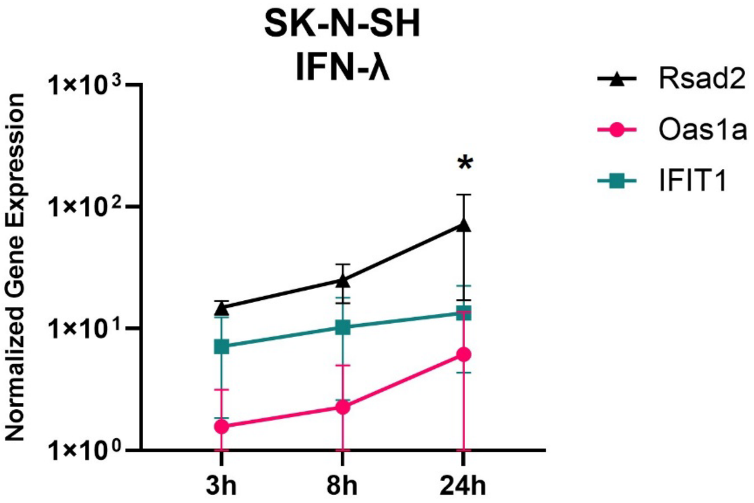

Supplement: Supplement 1 [file NIHPP2026.06.01.729199v1-supplement-1.pdf]
